# Fish biodiversity and assemblages along the altitudinal gradients of tropical mountainous forest streams

**DOI:** 10.1038/s41598-021-96253-3

**Published:** 2021-08-19

**Authors:** Chen-Lin Soo, Lee Nyanti, Nur Ezaimah Idris, Teck-Yee Ling, Siong-Fong Sim, Jongkar Grinang, Tonny Ganyai, Karen-Suan-Ping Lee

**Affiliations:** 1 Institute for Tropical Biology and Conservation, Universiti Malaysia Sabah, Jalan UMS, 88400 Kota Kinabalu, Sabah Malaysia; 2 Faculty of Resource Science and Technology, Universiti Malaysia Sarawak, 94300 Kota Samarahan, Sarawak Malaysia; 3 Research and Development Department, Sarawak Energy Berhad, 93050 Kuching, Sarawak Malaysia

**Keywords:** Biodiversity, Tropical ecology

## Abstract

Knowledge of the fundamental aspects of ecology such as the patterns of fish species distribution and biodiversity in the forest streams is the first and basic step to develop effective conservation strategies. Yet, studies on altitudinal changes of fish composition and assemblages in Bornean forest streams are scarce despite being one of the hotspots of biodiversity conservation. Hence, surveys on freshwater fish composition along the altitudinal gradients of the Baleh River Basin in Sarawak, Borneo were conducted from April 2014 to August 2015. The Baleh River Basin was divided into seven altitudinal groups with a total of 72 stations. Group elevation ranged from 53 to 269 m above sea level. The fish samples and environmental parameters were taken concurrently during samplings. A total of 3565 specimens belonging to six orders, 14 families, and 76 species were found in the present study. The most dominant family in the Baleh River Basin was Cyprinidae (74.4%), followed by Gastromyzontidae (16.2%) while the most dominant species was *Tor tambra* (12.9%), followed by *Lobocheilos ovalis* (12.3%). Fish abundance significantly higher at high altitude sites than those at low altitude sites except for Mengiong River which has the lowest fish abundance despite with high elevation. Species richness was found significantly lower in midstream segment. Noticeable altitudinal gradient of fish assemblages was observed along the Baleh River except a discontinuity at the midstream segment which is attributable to the poorer quality inflow from the Mengiong River coupled with the meandering feature of the segment. Fish abundance was significantly and positively correlated with elevation, water pH and conductivity while negatively correlated with turbidity. Anthropogenic activities in the Baleh River Basin had altered the environmental variables thus disrupted the altitudinal gradient of fish assemblages. This phenomenon is apparent when the Canonical Correspondence Analysis (CCA) revealed that the first axis (CCA1) explained 42.5% of the variation and has positive loading on dissolved oxygen (DO) and negative loading on water conductivity; whereas CCA2 explained 37.5% of the variation and positively loaded on elevation, water pH, and DO. The results demonstrated that *Gastromyzon fasciatus* preferred more oxygenated water than *Protomyzon sp., G. sp 1*, and *G. punctulatus* although they are all from Gastromyzontidae family that inhabiting high altitude sites. *Barbonymus schwanenfeldii* was also found most abundant with elevated dissolved oxygen value. On the other hand, *Rasbora volzii* and *R. hosii* inhabiting lower altitude sites with less oxygenated and more acidic water.

## Introduction

Altitudinal changes in the composition and biodiversity of fish fauna have been widely recorded^1–4^. Most of the studies covered a large scale of altitudinal gradient of fish assemblages, which are up to few thousand meters above sea level. For instance, the distinctiveness of the fish fauna had increased with elevation in streams of the central Andes of Colombia, where the greatest turnover was observed between 1000 and 1750 m above sea level, with nearly 90% of the species recorded between 250 and 1250 m above sea level^1^. Mercado-silva and Lyons investigated the fish assemblage patterns in a high gradient piedmont river in Mexico^2^. The authors demonstrated that sites with high altitude (> 1393 m) were dominated by non-native *Onchorhynchus mykiss* whereas higher species and guild diversity were found in sites with lower altitudes. However, a small scale of altitudinal gradient also exhibited effects on the patterns of fish assemblages. For instance, altitude was identified as one of the variables that had significant relationships with the fish assemblages in the North Tiaoxi River, China, located at an elevation ranging from 33 to 329 m^5^. Similarly, altitude was the main determinant of fish species richness and composition assemblages in streams of the Ivinhema River basin, Brazil, of which there were two zones of fish assemblages where species richness, size and composition in streams located above 430 m are different from streams below this altitude^6^.

Physical habitat and water quality variables are known as key components structuring fish assemblages^7,8^. Environmental conditions such as dissolved oxygen, water temperature, and water pH change with increasing elevation over short spatial distances in mountainous forest streams, thus affecting fish species distribution and biodiversity. Most fish communities experience losses of diversity with increasing altitude^3–6^, but not all respond to altitude in the same way where the trends could be peak at mid and high elevation or exhibited non-linear pattern^9–11^. The low diversity in high altitude sites is often attribute to the increasing harshness of environment conditions and to the decrease in the available habitat area. On the other hand, moderate environment condition and complex habitat diversity in the low altitude sites are among the factors contributing to higher diversity and richness in the downstream area^3^. However, other factors such as climate and anthropogenic activities in the local area could disrupt the altitudinal gradients as well^10,12^. For freshwater fishes, changes in environmental parameters could be deadly to them as they are flourish within specific optimum range and sensitive to the environmental changes^13,14^.

Knowledge of the fundamental aspects of ecology such as the patterns of fish species distribution and biodiversity in the forest streams is the first and basic step to develop effective conservation strategies. Yet, there is no known information on the composition and biodiversity of freshwater fish fauna and the factors affecting them within the altitudinal gradients of the Baleh River Basin in Sarawak, Borneo. The major activity in the vicinity is logging which could render the modification of water quality^15^ and subsequently alteration of fish distribution and biodiversity along the altitudinal gradients of the river^16,17^. Besides, a hydroelectric project has also been proposed at the Baleh River^18^ where dam construction could further alter water quality and environmental condition of the river when the project starts off^19,20^. Hence, the present study aimed to explore not only the composition and biodiversity of the fish fauna in the Baleh River Basin, but also to identify the altitudinal changes of fish abundance and biodiversity. We predicted that abundance and biodiversity of Bornean freshwater fish fauna varies along the altitudinal gradients of this mountainous forest streams in Sarawak. Lastly, we also investigated the relationship between fish species composition and selected environmental parameters within the altitudinal gradients of the Baleh River Basin. We predicted that there are key environmental factors that shape the fish community structure which would be useful for evaluating fish fauna with respect to their conservation management and for any potential mitigation measures if or where necessary, in the future.

## Materials and methods

### Study area

**Figure 1:**
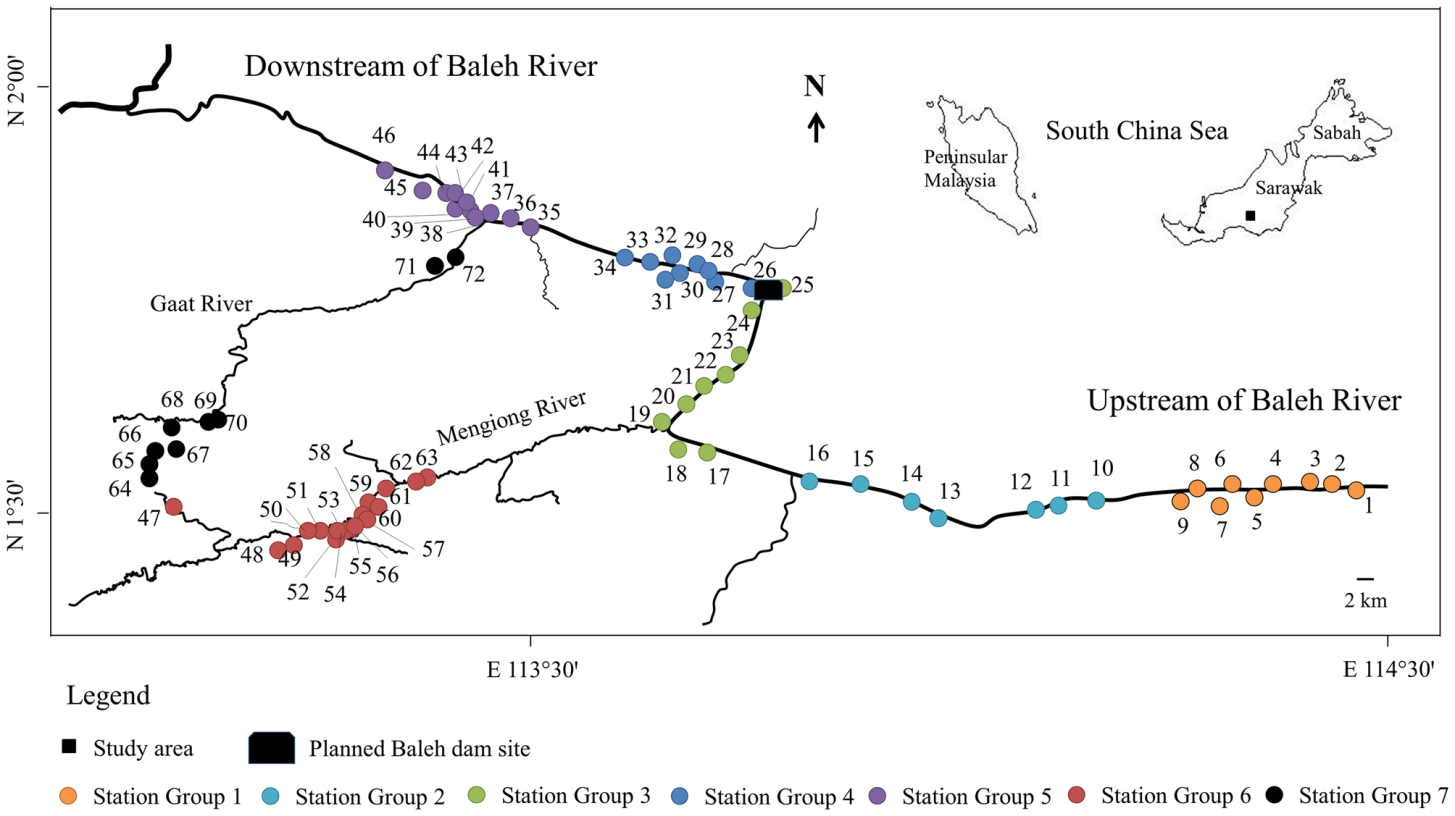
The location of the seven groups with a total of 72 sampling stations at the Baleh River Basin, Kapit, Sarawak.

Borneo forests are among the most biologically diverse habitats and act as a hotspot for biodiversity conservation. The Baleh River Basin is one of the mountainous forest streams in Sarawak, Malaysia, on the island of Borneo (Fig. 1). The forests are mostly characterized as mixed dipterocarp forest (MDF) and secondary forest^21^. The area is sparsely populated where the major activity in the vicinity is logging where signs of sedimentation problems in the river have been demonstrated^15^. A hydroelectric project has been proposed to build at the mid-stream of Baleh River (Fig. 1). Field samplings were conducted at a total of 72 stations located along the Baleh River and its two representative tributaries namely Mengiong River and Gaat River (Fig. 1). Majority of the sampling stations have been logged-over which result in a certain degree of habitat alterations. Sampling was conducted between April and August which corresponded with the dry season of the study area.

### Field protocols

Various fish sampling methods including electrofishing, cast net, three-layered nets, and monofilament gill nets of various mesh sizes were used. Electrofishing was done by using a portable electro-shocker (copper electrodes on wooden handles) that was powered by a portable AC generator. At each station, electrofishing was carried out from downstream to upstream at shallow, narrow, and fast flowing stretches for a distance of approximately 80 m for a duration of about 30 min. A scoop net (mesh size = 1 cm) was then used to collect the fishes. Cast net with a mesh size of 2.5 cm was thrown approximately 10 times at each station in the shallow water region of the stream. In addition, three-layered net (mesh size = 2.5, 7.6, and 12.7 cm) and monofilament gill net of different mesh sizes (mesh size, depth × length = 2.5 cm, 1.2 m × 6.0 m; 5.0 cm, 2.0 m × 10.0 m; 7.6 cm, 2.0 m × 10.0 m; 10.1 cm, 1.5 m × 12.0 m; and 12.7 cm, 2.0 m × 15.0 m) were placed at a deeper and wider part of the river and were left overnight. Nets were then checked at regular intervals of 5 h during the day. These methods were done at equal effort and distance at each station. The environmental parameters were measured concurrently with the sampling of fish. Elevation was taken using a Portable Global Positioning System (Garmin GPSmap 62sc). Temperature, dissolved oxygen (DO), turbidity, pH, and conductivity were measured from the surface water of streams at each station by using a multi-parameter Sonde (YSI 6920 V2-2). Fish were counted and identified in situ to the species level according to the taxonomic keys^22–26^ and confirmed with FishBase^27^ and Eschmeyer’s Catalog of Fishes^28^ for the latest taxonomic status. Samples that were unable to be identified in the field were fixed in 10% formalin and preserved in 70% ethanol for further identification in the laboratory.

### Data analysis

The 72 sampling stations were restructured into seven groups for data and statistical analysis (Table 1). Stations located along the Baleh River were grouped into five groups from upstream to downstream directions. Group 1 and Group 2 were located at the upstream of Baleh River. Group 3 and Group 4 were formed based on the location of the proposed dam site where Group 3 was located upstream of the proposed dam site and Group 4 was located below the dam site. Group 5 was located downstream of the Baleh River. The spatial distance between the groups of stations were approximately 10 km except for Group 3 and Group 4. Stations located at the two main tributaries namely Mengiong River and Gaat River were grouped as Group 6 and Group 7, respectively. Metrics of diversity including Shannon’s diversity index (H)^29^, Margalef index of species richness (D)^30^ and Pielou’s evenness (J)^31^ of fish at each station, each group of river segment, and the Baleh River Basin were determined by using the PAleontological STatistics software package (PAST, Palaeontological Association, 2001).

**Table 1:** Details of sampling regime and environmental parameters in the Baleh River Basin.

Group	Sampling station	N	Sampling date	Elevation (m a.s.l)	pH	Temperature (°C)	DO (mg/L)	Turbidity (NTU)	Conductivity (µS/cm)
1	St 1–St 9	9	April 2015	268.6 ± 42.2	7.6 ± 0.3	25.3 ± 1.0	7.9 ± 0.1	15.6 ± 12.2	44.4 ± 10.0
2	St 10–St 16	7	April 2015	176.6 ± 43.2	7.5 ± 0.2	26.0 ± 2.0	7.8 ± 0.3	13.6 ± 21.6	39.5 ± 7.3
3	St 17 and St 18	9	April 2015	135.0 ± 35.1	6.8 ± 0.5	26.8 ± 1.4	7.6 ± 0.3	7.0 ± 15.2	31.8 ± 11.5
	St 19–St 25		April 2014						
4	St 26–St 32	9	April 2014	71.6 ± 16.5	6.9 ± 0.4	25.3 ± 1.1	7.4 ± 0.6	39.0 ± 87.7	39.6 ± 15.2
	St 33 and St 34		August 2015						
5	St 35, St 39, St 42, St 44–St 46	12	August 2015	52.8 ± 23.0	7.2 ± 0.3	25.3 ± 1.0	7.3 ± 0.7	19.1 ± 41.1	38.8 ± 8.3
	St 36–St 38, St 40 and St 41		August 2014						
	St 43		April 2014						
6	St 47–St 63	17	June 2015	199.2 ± 25.3	7.1 ± 0.2	25.2 ± 1.1	7.5 ± 0.8	63.6 ± 67.8	26.0 ± 5.7
7	St 64–St 72	9	August 2014	182.8 ± 87.1	7.3 ± 0.3	26.2 ± 1.9	7.0 ± 0.9	6.1 ± 7.3	46.3 ± 17.4

A generalized linear mixed model (GLMM) analysis was performed to determine whether fish abundance and species richness were affected by elevation. In this analysis, data of fish abundance and species richness at each station was used as a response variable, whereas group type with a small scale of altitudinal gradients ranging from 53 to 269 m above sea level was treated as a fixed factor. To account statistically for the effects of differences in sampling time affecting the fish abundance and species richness at each station, sampling time was also included as a random factor in the model. Poisson was used as a family object and log link function was applied in the analysis. The Pearson correlation was conducted to identify environmental variables that affecting the abundance, species number, and metrics of diversity of freshwater fish along the altitudinal gradients of the Baleh River Basin by using data collected from the 72 sampling stations. The GLMM analysis and Pearson correlation were carried out by using the Statistical Software for Social Sciences (SPSS Version 26, SPSS Inc., 1995).

Finally, an ordination technique of Canonical Correspondence Analysis (CCA) was used in the direct analyses of the relationships between fish assemblages and environmental parameters^32^. Fifteen species that individually contributed more than 2% of the total fish caught and six environmental parameters were loaded in the CCA to study the relationships between fish assemblages and environmental parameters in the Baleh River Basin. The fish abundance data were standardized according to the number of stations at each group and Hellinger transformed prior for statistical analysis. The environmental parameters collected at each sampling station were pooled to determine the average value at each group. Table 1 summarizes the environmental parameters used in the analysis. Statistical significance (*p* value < 0.05) of the CCA relationships between the set of environmental factors and abundance of fish species was evaluated using a permutation test with 999 permutations^33^. CCA was carried out by using the PAleontological STatistics software package (PAST, Palaeontological Association, 2001).

## Results

### Freshwater fish composition and biodiversity in the Baleh River Basin

A total of 3565 specimens belonging to six orders, 14 families, and 76 species were captured in the Baleh River Basin (Table 2). Cyprinidae was the most dominant family (74.4%) in the Baleh River Basin. The most dominant species was *Tor tambra* (12.9%) and *Lobocheilos ovalis* (12.3%), followed by *Rasbora volzii* (7.1%), *Crossocheilus cobitis* (5.8%), and *Rasbora borneensis* (5.5%), all of which are from the family Cyprinidae. On the other hand, family Gastromyzontidae was the second most dominant family (16.2%) in the Baleh River Basin where *Parhomaloptera microstoma* (3.5%) was the most dominant fish species. The metrics of diversity including Shannon’s diversity index (H), Margalef index of species richness (D), and Pielou’s evenness (J) in the Baleh River Basin and each group of river segment were summarized in Table 2.

**Table 2:** Summary of the fish composition and metrics of diversity in the Baleh River Basin.

Species	Group 1		Group 2		Group 3		Group 4		Group 5		Group 6		Group 7		Baleh River Basin	
	N	%	N	%	N	%	N	%	N	%	N	%	N	%	N	%
**Order: Anabantiformes**																
Family: Channidae																
* Channa lucius*	0	0.0	0	0.0	0	0.0	4	1.1	4	1.0	2	0.5	1	0.1	11	0.3
* Channa striata*	0	0.0	0	0.0	0	0.0	0	0.0	5	1.2	1	0.2	1	0.1	7	0.2
Sub-total number of species	0	0.0	0	0.0	0	0.0	1	2.4	2	4.4	2	6.5	2	5.9	2	2.6
Sub-total number of fish caught	0	0.0	0	0.0	0	0.0	4	1.1	9	2.2	3	0.7	2	0.2	18	0.5
**Order: Cypriniformes**																
Family: Balitoridae																
* Balitoropsis zollingeri*	0	0.0	1	0.3	0	0.0	0	0.0	0	0.0	0	0.0	0	0.0	1	0.0
* Homaloptera ogilviei*	0	0.0	0	0.0	0	0.0	0	0.0	0	0.0	0	0.0	6	0.6	6	0.2
* Homaloptera orthogoniata*	2	0.3	0	0.0	2	0.7	2	0.5	0	0.0	0	0.0	2	0.2	8	0.2
* Homalopteroides tweediei*	0	0.0	3	0.8	0	0.0	0	0.0	5	1.2	1	0.2	3	0.3	12	0.3
* Homalopteroides wassinkii*	0	0.0	2	0.5	0	0.0	0	0.0	0	0.0	0	0.0	0	0.0	2	0.1
Sub-total number of species	1	3.4	3	9.1	1	3.7	1	2.4	1	2.2	1	3.2	3	8.8	5	6.4
Sub-total number of fish caught	2	0.3	6	1.6	2	0.7	2	0.5	5	1.2	1	0.2	11	1.2	29	0.8
Family: Cyprinidae																
* Cyclocheilichthys repasson*	0	0.0	0	0.0	0	0.0	2	0.5	0	0.0	0	0.0	0	0.0	2	0.1
* Barbodes kuchingensis*	0	0.0	0	0.0	0	0.0	19	5.1	15	3.6	8	1.8	10	1.1	52	1.5
* Barbodes lateristriga*	0	0.0	0	0.0	0	0.0	14	3.8	12	2.9	0	0.0	11	1.2	37	1.0
* Barbonymus balleroides*	0	0.0	0	0.0	0	0.0	5	1.4	0	0.0	4	0.9	0	0.0	9	0.3
* Barbonymus collingwoodii*	3	0.4	48	12.9	5	1.7	10	2.7	5	1.2	34	7.7	32	3.4	137	3.8
* Barbonymus schwanenfeldii*	5	0.7	34	9.1	23	7.7	10	2.7	19	4.6	29	6.6	0	0.0	120	3.4
* Crossocheilus cobitis*	64	8.8	9	2.4	0	0.0	5	1.4	0	0.0	32	7.3	97	10.2	207	5.8
* Crossocheilus oblongus*	0	0.0	0	0.0	0	0.0	0	0.0	0	0.0	0	0.0	18	1.9	18	0.5
* Cyclocheilichthys apogon*	2	0.3	4	1.1	0	0.0	5	1.4	18	4.4	19	4.3	9	0.9	57	1.6
* Cyclocheilichthys armatus*	2	0.3	3	0.8	24	8.1	4	1.1	8	1.9	0	0.0	6	0.6	47	1.3
* Cyprinus carpio*	0	0.0	0	0.0	0	0.0	0	0.0	1	0.2	0	0.0	0	0.0	1	0.0
* Hampala bimaculata*	0	0.0	7	1.9	3	1.0	5	1.4	6	1.5	19	4.3	9	0.9	49	1.4
* Hampala macrolepidota*	0	0.0	0	0.0	0	0.0	9	2.4	4	1.0	0	0.0	1	0.1	14	0.4
* Labiobarbus fasciatus*	3	0.4	1	0.3	0	0.0	11	3.0	0	0.0	4	0.9	0	0.0	19	0.5
* Labiobarbus festivus*	0	0.0	0	0.0	3	1.0	0	0.0	0	0.0	1	0.2	0	0.0	4	0.1
* Labiobarbus leptocheilus*	0	0.0	0	0.0	3	1.0	1	0.3	0	0.0	0	0.0	0	0.0	4	0.1
* Lobocheilos ovalis*	129	17.8	61	16.4	68	22.8	35	9.5	6	1.5	61	13.9	79	8.3	439	12.3
* Lobocheilos schwanenfeldii*	0	0.0	0	0.0	0	0.0	0	0.0	1	0.2	0	0.0	0	0.0	1	0.0
* Lobocheilos* cf. *falcifer*	22	3.0	46	12.4	0	0.0	4	1.1	1	0.2	18	4.1	14	1.5	105	2.9
* Luciosoma setigerum*	3	0.4	26	7.0	0	0.0	1	0.3	2	0.5	34	7.7	1	0.1	67	1.9
* Luciosoma spilopleura*	0	0.0	0	0.0	4	1.3	3	0.8	3	0.7	6	1.4	0	0.0	16	0.4
* Luciosoma trinema*	0	0.0	0	0.0	0	0.0	1	0.3	1	0.2	0	0.0	0	0.0	2	0.1
* Nematabramis sp.*	0	0.0	0	0.0	0	0.0	0	0.0	1	0.2	0	0.0	0	0.0	1	0.0
* Osteochilus schlegelii*	1	0.1	1	0.3	3	1.0	10	2.7	9	2.2	1	0.2	2	0.2	27	0.8
* Osteochilus vittatus*	9	1.2	12	3.2	19	6.4	11	3.0	23	5.6	10	2.3	33	3.5	117	3.3
* Osteochilus waandersii*	0	0.0	0	0.0	0	0.0	0	0.0	1	0.2	0	0.0	0	0.0	1	0.0
* Osteochilus melanopleurus*	0	0.0	0	0.0	0	0.0	0	0.0	0	0.0	13	3.0	0	0.0	13	0.4
* Oxygaster anomalura*	5	0.7	17	4.6	0	0.0	0	0.0	0	0.0	28	6.4	0	0.0	50	1.4
* Paracrossochilus acerus*	0	0.0	0	0.0	4	1.3	1	0.3	0	0.0	0	0.0	0	0.0	5	0.1
* Puntioplites waandersi*	0	0.0	0	0.0	0	0.0	8	2.2	0	0.0	0	0.0	0	0.0	8	0.2
* Rasbora bankanensis*	0	0.0	0	0.0	0	0.0	0	0.0	5	1.2	0	0.0	0	0.0	5	0.1
* Rasbora borneensis*	67	9.2	13	3.5	1	0.3	0	0.0	74	18.0	17	3.9	25	2.6	197	5.5
* Rasbora volzii*	0	0.0	1	0.3	3	1.0	93	25.1	57	13.8	12	2.7	88	9.3	254	7.1
* Rasbora hosii*	0	0.0	4	1.1	7	2.3	19	5.1	30	7.3	0	0.0	33	3.5	93	2.6
* Tor tambra*	155	21.4	34	9.1	86	28.9	14	3.8	8	1.9	38	8.6	126	13.3	461	12.9
* Tor tambroides*	15	2.1	0	0.0	0	0.0	0	0.0	0	0.0	0	0.0	0	0.0	15	0.4
Sub-total number of species	15	51.7	17	51.5	16	59.3	25	61.0	24	53.3	20	64.5	18	52.9	37	47.4
Sub-total number of fish caught	485	66.9	321	86.3	256	85.9	300	81.1	310	75.2	388	88.2	594	62.7	2654	74.4
Family: Gastromyzontidae																
* Gastromyzon sp 1*	6	0.8	0	0.0	0	0.0	0	0.0	0	0.0	3	0.7	78	8.2	87	2.4
* Gastromyzon sp 2*	1	0.1	0	0.0	3	1.0	0	0.0	0	0.0	0	0.0	22	2.3	26	0.7
* Gastromyzon fasciatus*	62	8.6	10	2.7	0	0.0	0	0.0	0	0.0	17	3.9	0	0.0	89	2.5
* Gastromyzon punctulatus*	7	1.0	8	2.2	0	0.0	0	0.0	0	0.0	0	0.0	83	8.8	98	2.7
* Gastromyzon sp 3*	0	0.0	0	0.0	0	0.0	0	0.0	0	0.0	0	0.0	2	0.2	2	0.1
* Neogastromyzon chini*	17	2.3	0	0.0	0	0.0	0	0.0	0	0.0	0	0.0	0	0.0	17	0.5
* Neogastromyzon nieuwenhuisii*	8	1.1	0	0.0	0	0.0	0	0.0	0	0.0	0	0.0	0	0.0	8	0.2
* Neogastromyzon pauciradiatus*	37	5.1	0	0.0	0	0.0	0	0.0	0	0.0	0	0.0	0	0.0	37	1.0
* Parhomaloptera microstoma*	59	8.1	3	0.8	10	3.4	7	1.9	0	0.0	9	2.0	35	3.7	123	3.5
* Protomyzon sp.*	0	0.0	0	0.0	1	0.3	0	0.0	0	0.0	0	0.0	88	9.3	89	2.5
Sub-total number of species	8	27.6	3	9.1	3	11.1	1	2.4	0	0.0	3	9.7	6	17.6	10	12.8
Sub-total number of fish caught	197	27.2	21	5.6	14	4.7	7	1.9	0	0.0	29	6.6	308	32.5	576	16.2
Family: Nemacheilidae																
* Nemacheilus kapuasensis*	0	0.0	1	0.3	3	1.0	14	3.8	5	1.2	0	0.0	1	0.1	24	0.7
* Nemacheilus sp.*	0	0.0	0	0.0	0	0.0	0	0.0	1	0.2	0	0.0	0	0.0	1	0.0
* Nemacheilus spiniferus*	6	0.8	0	0.0	1	0.3	0	0.0	0	0.0	1	0.2	0	0.0	8	0.2
Sub-total number of species	1	3.4	1	3.0	2	7.4	1	2.4	2	4.4	1	3.2	1	2.9	3	3.8
Sub-total number of fish caught	6	0.8	1	0.3	4	1.3	14	3.8	6	1.5	1	0.2	1	0.1	33	0.9
**Order: Perciformes**																
Family: Ambassidae																
* Parambassis wolffii*	0	0.0	0	0.0	0	0.0	0	0.0	6	1.5	0	0.0	0	0.0	6	0.2
Sub-total number of species	0	0.0	0	0.0	0	0.0	0	0.0	1	2.2	0	0.0	0	0.0	1	1.3
Sub-total number of fish caught	0	0.0	0	0.0	0	0.0	0	0.0	6	1.5	0	0.0	0	0.0	6	0.2
**Order: Siluriformes**																
Family: Akysidae																
* Acrochordonichthys ischnosoma*	0	0.0	0	0.0	0	0.0	0	0.0	3	0.7	0	0.0	0	0.0	3	0.1
* Acrochordonichthys rugosus*	0	0.0	2	0.6	0	0.0	0	0.0	0	0.0	0	0.0	0	0.0	2	0.0
Sub-total number of species	0	0.0	2	6.1	0	0.0	0	0.0	1	2.2	0	0.0	0	0.0	3	3.8
Sub-total number of fish caught	0	0.0	2	0.5	0	0.0	0	0.0	3	0.7	0	0.0	0	0.0	5	0.1
Family: Bagridae																
* Hemibagrus fortis*	0	0.0	0	0.0	0	0.0	0	0.0	2	0.5	0	0.0	0	0.0	2	0.1
* Hemibagrus capitulum*	6	0.8	6	1.6	10	3.4	7	1.9	10	2.4	12	2.7	3	0.3	54	1.5
* Hemibagrus wyckii*	0	0.0	1	0.3	0	0.0	1	0.3	0	0.0	0	0.0	0	0.0	2	0.1
* Leiocassis micropogon*	3	0.4	7	1.9	1	0.3	0	0.0	2	0.5	0	0.0	0	0.0	13	0.4
* Leiocassis poecilopterus*	0	0.0	0	0.0	0	0.0	0	0.0	1	0.2	0	0.0	0	0.0	1	0.0
* Mystus singaringan*	0	0.0	2	0.5	0	0.0	3	0.8	0	0.0	0	0.0	0	0.0	5	0.1
Sub-total number of species	2	6.9	4	12.1	2	7.4	3	7.3	4	8.9	1	3.2	1	2.9	6	7.7
Sub-total number of fish caught	9	1.2	16	4.3	11	3.7	11	3.0	15	3.6	12	2.7	3	0.3	77	2.2
Family: Clariidae																
Clarias leiacanthus	0	0.0	0	0.0	0	0.0	2	0.5	0	0.0	0	0.0	2	0.2	4	0.1
Sub-total number of species	0	0.0	0	0.0	0	0.0	1	2.4	0	0.0	0	0.0	1	2.9	1	1.3
Sub-total number of fish caught	0	0.0	0	0.0	0	0.0	2	0.5	0	0.0	0	0.0	2	0.2	4	0.1
Family: Pangasiidae																
* Pangasius macronema*	0	0.0	2	0.5	1	0.3	6	1.6	4	1.0	1	0.2	0	0.0	14	0.4
* Pangasius nieuwenhuisii*	0	0.0	0	0.0	0	0.0	3	0.8	4	1.0	0	0.0	0	0.0	7	0.2
* Pseudolais micronemus*	0	0.0	0	0.0	0	0.0	6	1.6	12	2.9	0	0.0	0	0.0	18	0.5
Sub-total number of species	0	0.0	1	3.0	1	3.7	3	7.3	3	6.7	1	3.2	0	0.0	3	3.8
Sub-total number of fish caught	0	0.0	2	0.5	1	0.3	15	4.1	20	4.9	1	0.2	0	0.0	39	1.1
Family: Siluridae																
* Kryptopterus bicirrhis*	0	0.0	0	0.0	0	0.0	0	0.0	3	0.7	0	0.0	0	0.0	3	0.1
* Kryptopterus lais*	0	0.0	1	0.3	0	0.0	4	1.1	8	1.9	0	0.0	0	0.0	13	0.4
* Kryptopterus limpok*	0	0.0	0	0.0	0	0.0	1	0.3	3	0.7	0	0.0	0	0.0	4	0.1
* Phalacronotus apogon*	0	0.0	0	0.0	0	0.0	3	0.8	1	0.2	0	0.0	0	0.0	4	0.1
Sub-total number of species	0	0.0	1	3.0	0	0.0	3	7.3	4	8.9	0	0.0	0	0.0	4	5.1
Sub-total number of fish caught	0	0.0	1	0.3	0	0.0	8	2.2	15	3.6	0	0.0	0	0.0	24	0.7
Family: Sisoridae																
* Glyptothorax platypogonides*	25	3.4	2	0.5	9	3.0	6	1.6	1	0.2	4	0.9	17	1.8	64	1.8
Sub-total number of species	1	3.4	1	3.0	1	3.7	1	2.4	1	2.2	1	3.2	1	2.9	1	1.3
Sub-total number of fish caught	25	3.4	2	0.5	9	3.0	6	1.6	1	0.2	4	0.9	17	1.8	64	1.8
**Order: Synbranchiformes**																
Family: Mastacembelidae																
* Macrognathus maculatus*	1	0.1	0	0.0	1	0.3	0	0.0	11	2.7	1	0.2	10	1.1	24	0.7
Sub-total number of species	1	3.4	0	0.0	1	3.7	0	0.0	1	2.2	1	3.2	1	2.9	1	1.3
Sub-total number of fish caught	1	0.1	0	0.0	1	0.3	0	0.0	11	2.7	1	0.2	10	1.1	24	0.7
**Order: Tetraodontiformes**																
Family: Tetraodontidae																
* Auriglobus modestus*	0	0.0	0	0.0	0	0.0	1	0.3	11	2.7	0	0.0	0	0.0	12	0.3
Sub-total number of species	0	0.0	0	0.0	0	0.0	1	2.4	1	2.2	0	0.0	0	0.0	1	1.3
Sub-total number of fish caught	0	0.0	0	0.0	0	0.0	1	0.3	11	2.7	0	0.0	0	0.0	12	0.3
Total number of species	29		32		26		41		45		31		34		76	
Total number of fish caught	725		372		298		370		412		440		948		3565	
Total number of stations	9		7		9		9		12		17		9		72	
Abundancy (ind/st)	81		54		34		42		35		26		106		50	
Shannon’s diversity index, H (e)	2.5		2.8		2.4		3.1		3.1		3.0		2.9		3.4	
Margalef’s richness index, D (e)	4.3		5.2		4.4		6.8		7.3		4.9		4.8		9.2	
Pielou’s evenness index, J (e)	0.8		0.8		0.7		0.8		0.8		0.9		0.8		0.8	

### Altitudinal changes of freshwater fish in the Baleh River Basin

Generalized linear mixed model (GLMM) demonstrated that elevation significantly influences fish abundance (*p* value < 0.001) in the Baleh River basin after statistically controlling for differences in sampling time as random variable. Significantly higher fish abundance was found in Group 1 and Group 2 that located at upstream (positive coefficient values range: 0.621 to 1.037, *p* value < 0.05, Table 3) whereas significantly lower fish abundance was found in Groups 3, 4, and 5 that located at downstream (negative coefficient values range: − 1.011 to − 0.989, *p* value < 0.05, Table 3) as compared to Group 7 (as reference). On the other hand, species richness at Group 3 (negative coefficient value: − 2.188, *p* value = 0.032, Table 3) was significantly low in the Baleh River Basin. Fish abundance of the Baleh River Basin was also found to be significantly and positively correlated with elevation, water pH, and conductivity, and negatively correlated with turbidity (Table 4, *p* value < 0.05). In contrast to fish abundance, no significant correlations (*p* value ≥ 0.05) between environmental variables and species richness or metrics of diversity were found in the present study.

**Table 3:** Generalized linear mixed-effects model (GLMM) for the effect of elevation on fish abundance and species richness (n = 72).

Variable	Fixed effects					Fixed coefficients					Random effect covariance			
	Source	F	df1	df2	Sig.	Model term	Coefficient	Std. error	t	Sig.	Estimate	Std. error	Z	Sig.
Fish abundance	Corrected model (Group)	52.292	6	65	** < 0.001**	Intercept	4.274	0.3403	12.559	** < 0.001**	0.410	0.370	1.109	0.268
						Group 1	1.037	0.2702	3.838	** < 0.001**				
						Group 2	0.621	0.2726	2.279	**0.026**				
						Group 3	− 0.989	0.1763	− 5.607	** < 0.001**				
						Group 4	− 0.900	0.1571	− 5.731	** < 0.001**				
						Group 5	− 1.011	0.0733	− 13.792	** < 0.001**				
						Group 6	− 1.021	0.7269	− 1.404	0.165				
						Group 7	Reference							
Species richness	Corrected model (Group)	2.396	6	65	**0.037**	Intercept	2.354	0.2221	10.600	** < 0.001**	0.076	0.088	0.861	0.389
						Group 1	− 0.126	0.3299	− 0.381	0.705				
						Group 2	0.153	0.3293	0.464	0.644				
						Group 3	− 0.631	0.2886	− 2.188	**0.032**				
						Group 4	− 0.306	0.2678	− 1.143	0.257				
						Group 5	0.009	0.1497	0.061	0.952				
						Group 6	− 0.592	0.3676	− 1.610	0.112				
						Group 7	Reference							

Sampling time was included as a random factor in the model.

Significant value (*p* value < 0.05) was indicated in bold.

**Table 4:** Pearson correlation analysis between fish biodiversity and environmental parameters (n = 72).

	Elevation	pH	Temperature	DO	Turbidity	Conductivity	Abundance	Species number	H	D	J
Elevation	1.000										
pH	**0.338**	1.000									
Temperature	− 0.103	0.007	1.000								
DO	0.107	0.085	− **0.289**	1.000							
Turbidity	0.036	− 0.014	− 0.020	− 0.089	1.000						
Conductivity	0.028	**0.372**	0.163	0.053	− **0.313**	1.000					
Abundance	**0.334**	**0.276**	− 0.019	− 0.055	− **0.246**	**0.346**	1.000				
Species number	− 0.093	0.110	− 0.028	− 0.039	− 0.171	0.214	**0.543**	1.000			
H	− 0.097	0.038	− 0.062	− 0.109	− 0.226	0.138	**0.414**	**0.891**	1.000		
D	− 0.223	− 0.007	− 0.009	− 0.087	− 0.060	0.062	0.228	**0.916**	**0.899**	1.000	
J	0.096	0.011	− 0.033	− 0.221	0.109	− 0.210	− 0.205	0.063	**0.371**	**0.282**	1.000

Significant value (*p* value < 0.05) was indicated in bold.

### Fish assemblages of the Baleh River Basin associated with environmental parameters

**Figure 2:**
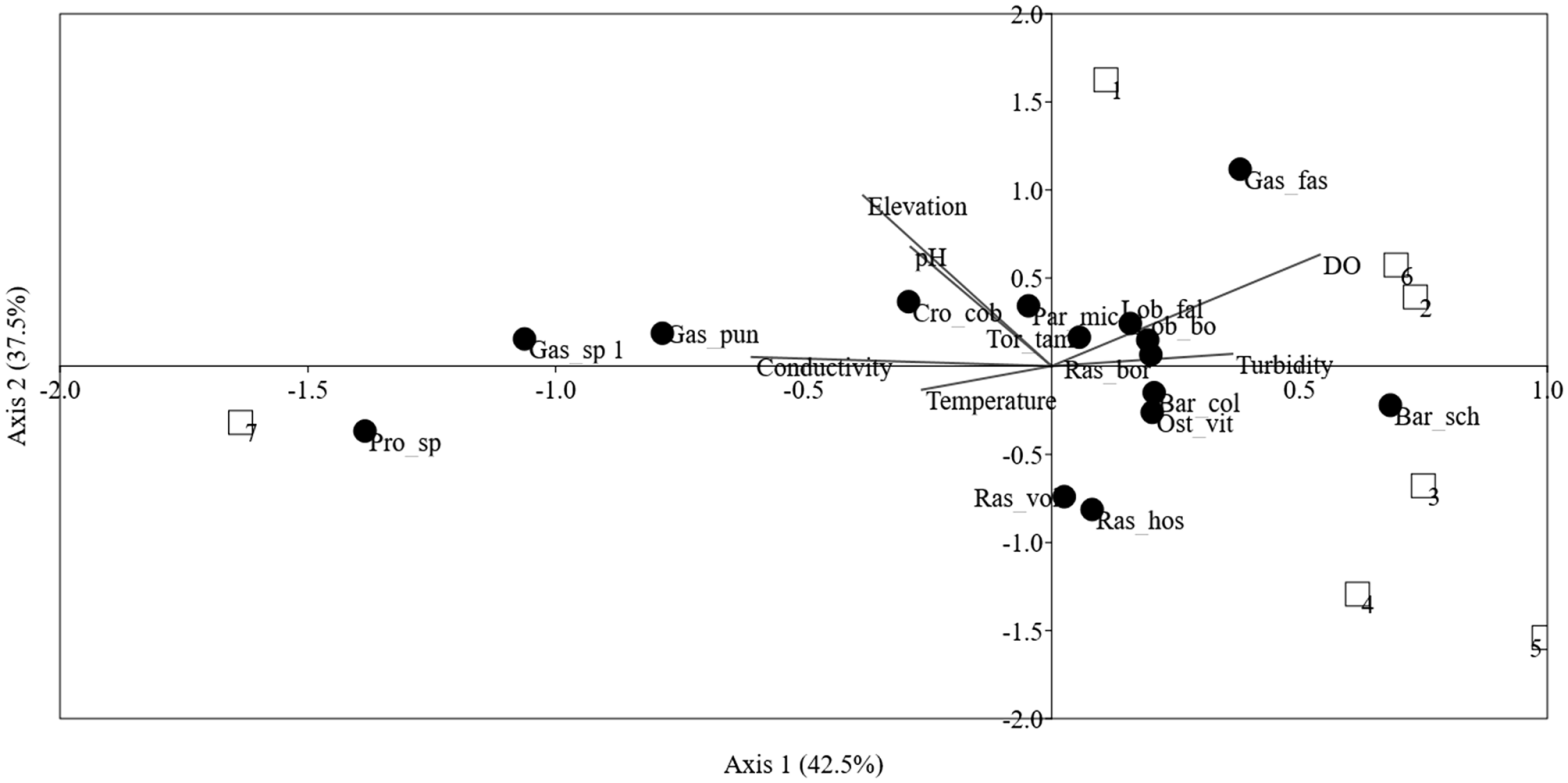
CCA ordination triplots of the abundance of the 15 species (> 2%) with six environmental parameters at seven groups in the Baleh River Basin. Bar_col = *Barbonymus collingwoodii*, Bar_sch = *Barbonymus schwanenfeldii*, Cro_cob = *Crossocheilus cobitis*, Lob_ova = *Lobocheilos ovalis*, Lob_fal = *Lobocheilos* cf. *falcifer*, Ost_vit = *Osteochilus vittatus*, Ras_bor = *Rasbora borneensis*, Ras_vol = *Rasbora volzii*, Ras_hos = *Rasbora hosii*, Tor_tam = *Tor tambra*, Gas_sp 1 = *Gastromyzon sp 1*, Gas_fas = *Gastromyzon fasciatus*, Gas_pun = *Gastromyzon punctulatus*, Par_mic = *Parhomaloptera microstama*, Pro_sp. = *Protomyzon sp.*

The first CCA axis (CCA1) had an eigenvalue of 0.219 and explained 42.5% of the species-environment relation variance (Fig. 2). The CCA2 accounted for 37.5% of total variance with an eigenvalue of 0.193. These two axes were statistically significant (*p* value < 0.05) according to the Monte-Carlo permutation test. The CCA1 was positively correlated with DO but negatively correlated with conductivity. On the other hand, the elevation, water pH, and DO were found positively loaded on CCA2. Gastromyzonid fishes were highly loaded on CCA1 but exhibited different responses. *Protomyzon sp.*, *Gastromyzon sp 1*, and *G. punctulatus* were negatively loaded on CCA1 whereas *G. fasciatus* was positively loaded on CCA1. One cyprinid, *Barbonymus schwanenfeldii* was positively loaded on CCA 1. *Gastromyzon fasciatus* was positively loaded on CCA2 whereas two cyprinids (*Rasbora volzii* and *Rasbora hosii*) had high negative loadings on CCA2. Figure 2 illustrates that fish assemblages of the Baleh River Basin varied according to groups. Groups 1, 2, and 6 were located at upper right portion of the CCA ordination triplot whereas Groups 3, 4, and 5 were located at lower right portion of the triplot. Group 7 was located at the lower left portion of the CCA ordination triplot.

## Discussion

The present study demonstrated that the most dominant family in the Baleh River Basin, Sarawak was Cyprinidae followed by Gastromyzontidae. Dominance by the family Cyprinidae (74.4%) in this tropical forest stream is common due to their highly morphological adaptations^34^ and is corroborated by previous studies^35–38^. The family Gastromyzontidae that was resurrected by Kottelat^39^ in his revision of the loaches was the second most dominant family (16.2%) in the Baleh River Basin. Gastromyzontidae (sucker loaches), Balitoridae (hill stream loaches), and Nemacheilidae (stone loaches) are families of loaches which are small and elongated bottom-dwelling freshwater fishes with several barbels near the mouth. Most species are rheophilic, living in the headwater and hill streams, typically in fast-flowing water over a rocky substratum and high elevation mountainous areas^40^. They are found mostly at high altitude mountainous areas^38,41^. Hence, it is not surprising that these families contribute up to 28.3% and 33.8% at the upstream segment of the Baleh River (Group 1) and upstream of the Gaat River (Group 7), respectively (Table 2). Similarly, the Balitoridae contributed 36% to the fish fauna composition in the headwater of Ulu Tungud Forest Reserve, Meliau, Sabah, and ranked as the second most dominant family in the study area^42^. The loaches family (Balitoridae and Nemacheilidae) also contributed 20.59% of fish composition in the Upper Pelus River, Perak^17^. Overall, the fish diversity in the Baleh River Basin was observed to be moderate with a Shannon’s diversity index of 3.4^43^ while the species richness with an index value of 9.2 was high^44^. The fish assemblages were evenly spread in the Baleh River Basin with an evenness index of 0.8^31^.

The fish abundance in the Baleh River Basin was significantly influenced by elevation (*p* value < 0.05) where fish abundance steadily increased as altitude increased except for Mengiong River (Group 6). The high fish abundance in upstream river of the Baleh River and Gaat River is attributable to a better water quality in upstream as indicated by the positive correlation of fish abundance with elevation, pH, and conductivity, and negative correlation of fish abundance with turbidity. On the other hand, water quality of the Mengiong River is comparatively poorer with lower water pH and conductivity, and higher turbidity than those rivers at similar elevation. The present study areas are subjected to logging activities that leads to the low water pH and high turbidity^15^. Turbid condition in water can influence freshwater fishes in many ways, such as causing physical damage to gill structure of fish and clogging which leads to respiratory failure, and decreasing anti predator behavior, foraging efficiency, and hatching rate of fish^45–47^. In addition, sedimentation and degradation of stream substrates can diminish the food base for benthic fish species such as loaches^48^. Quality of the environment affects fish abundance and reproductive rate^49–51^ thus it might not be linearly correlated with elevation. A unimodal response of total fish abundance was also observed in the European part of Russia where the highest fish numbers were found at elevations between 250 and 500 m^50^.

Fish species richness in the present study did not follow the well documented trend of species richness decrease with altitude, instead significantly low fish species richness (*p* value < 0.05) was observed at Group 3. Group 3 was located at the midstream segment of the Baleh River that follows a sinuous trail through the basin. It was consistently possessing lowest values in fish abundance and metrics of diversity between the five groups located in the Baleh River, despite the spatial structure of the fish assemblages along the Baleh River demonstrating noticeable upstream–downstream gradient. The discontinuity of the upstream–downstream gradient at the midstream segment is most probably due to the influence of the inflow water from the Mengiong River coupled with the meandering feature of the segment. The midstream segment is receiving the inflow water of the Mengiong River which contains high organic matter and suspended solids. The meandering feature of the midstream segment decreases water velocity at the segment contributing to the lower turbidity at the area. However, it also increases accumulation of organic matter which is evidenced by the low conductivity value and acidic pH value at the midstream segment (Table 1). Low water pH has been shown to have detrimental effects on fish growth and physiological responses^52,53^. Hence, the low abundance and metrics of diversity in the midstream segment are most probably attributable to the acidic water in the river. Previous literature also showed that species richness can respond to altitude in many other ways. Fish species richness could be peaked at mid-elevation as seen in the Tibetan Plateau and its adjacent regions^10^. Higher species richness was observed in upstream rivers of the Tayabas River in Philippines where the authors attributed the phenomenon to the effect of anthropogenic disturbances at the downstream area^54^.

Fish species can prefer aquatic habitats with specific requirements, such as *Gastromyzon fasciatus* and *Barbonymus schwanenfeldii* that were found to be highly associated with elevated values of dissolved oxygen in the present study. In fact, the most important environmental parameters structuring the fish assemblage in the Baleh River Basin were dissolved oxygen and conductivity as indicated by their high loadings on CCA1. Dissolved oxygen in water is one of the most important factors in water quality where hypoxia is a common cause of fish kills around the world^55^. Fish abundance and diversity are highly associated with dissolved oxygen in river water^7^. Besides, conductivity has also been shown to be an important factor associated with fish composition and assemblages in aquatic ecosystem^7,56^. Stream conductivity is commonly associated with dissolved ions which are primarily affected by the geology of the area where the water flows through. In addition, conductivity could reflect anthropogenic interactions as it had been demonstrated that compositional differences between clear-felled, buffered (100 m width) and unlogged streams were related to the abundance of fine and coarse particulate organic matter and conductivity^16^. Also, the conductivity value decreased after the surrounding trees were harvested in wetlands of Georgia^57^. The lowest fish abundance at Mengiong River coincided with the lowest conductivity and the highest turbidity suggesting that high organic matter and suspended solids in the river due to logging activities have reduced fish abundance in the river. Organic compounds do not conduct electrical current well, hence the low conductivity value when organic matter is high in the stream. On the other hand, higher fish abundance is associated with higher values of conductivity at the upstream segments of the Baleh River and the Gaat River. The high conductivity value at those segments is probably attributable to its unique geological characteristic and marginally anthropogenic impact. Similarly, Cheimonopoulou et al.^58^ attributed the higher values of conductivity, hardness, and pH at the minimally impacted station from a small Mediterranean river to its unique geology and/or to low discharge values.

It has no doubt that elevation was one of the variables that had significant relationship with the fish assemblages in the Baleh River Basin despite the exception in the Mengiong River. The present study demonstrates that fishes from Gastromyzontidae family are common in upper stretches of river with higher elevation, which in this case are the upper segment of Baleh River. Fishes from Gastromyzontidae family were previously grouped as Balitoridae family which are often known as hill stream loaches due to their ability to cope with fast flowing water^59^. Previous study demonstrated that *Gastromyzon* spp. has morphologically adapted to live in torrential waters by clinging tightly to solid surfaces^60^ and were found restricted to headwater^61^. Hence, it is not surprising that those fishes were found proliferating in the hilly area at the Baleh River Basin. Nevertheless, *Gastromyzon fasciatus* is separated from other gastromyzonid species by its specific requirement of well-oxygenated waters and elevated water pH. The fish was found abundantly at the upstream segments of Baleh River (Group 1 and Group 2) and Mengiong River (Group 6) that positively loaded on CCA1 as those groups are high in DO values (≈ 7.73 mg/L). On the other hand, *Protomyzon sp.*, *Gastromyzon sp 1*, and *G. punctulatus* have no specific requirement on high DO value. This is most apparent when they were found most abundantly or restricted at the Gaat River where the DO content (≈ 7.0 ± 0.9) was the lowest among the stream segments. The distinctive habitat associations among gastromyzonids are not uncommon. Some balitorid species in Thailand are also geographically separated, while others share similar distributions^41^. In contrast to gastromyzonids, two cyprinids from genus *Rasbora* were found mostly inhabiting in streams with lower elevation and water pH.

## Conclusions

A total of 3565 specimens belonging to six orders, 14 families, and 76 species were collected at different altitudes at the Baleh River Basin. Cyprinidae was the most dominant family (74.4%) in the Baleh River Basin, followed by Gastromyzontidae (16.2%). Overall, the fish composition in the Baleh River Basin is rich in species, moderately diverse, and spread evenly along the river. The fish abundance increased significantly with increased altitude although the altitudinal gradient was disrupted at the midstream segment most probably due to the meandering feature of the midstream segment and the inflow from the Mengiong tributary. Elevation plays an important role in structuring fish assemblages in the Baleh River Basin that located at an elevation ranging from 53 to 269 m above sea level. However, anthropogenic activities had altered the environmental variables in the area. Thus, fish assemblages in the Baleh River Basin were found highly associated with DO and conductivity. Gastromyzonids which are characteristically present in more turbulent habitats are common in upper stretches of the river with higher elevation. The present study demonstrates that gastromyzonids could have similar habitat associations or be geographically separated in the Baleh River Basin. In contrast to gastromyzonids, cyprinids like *Rasbora volzii* and *R. hosii* were found inhabiting streams with lower elevation.
